# Effect of High-Pressure Processing on Physico-Chemical, Microbiological and Sensory Traits in Fresh Fish Fillets (*Salmo salar* and *Pleuronectes platessa*)

**DOI:** 10.3390/foods10081775

**Published:** 2021-07-30

**Authors:** Marta Castrica, Radmila Pavlovic, Claudia M. Balzaretti, Giulio Curone, Gabriele Brecchia, Emma Copelotti, Sara Panseri, Davide Pessina, Claudio Arnoldi, Luca Maria Chiesa

**Affiliations:** 1 Department of Health, Animal Science and Food Safety “Carlo Cantoni”, Università degli Studi di Milano, Via dell’Università 6, 26900 Lodi, Italy; marta.castrica@unimi.it (M.C.); radmila.pavlovic1@unimi.it (R.P.); claudia.balzaretti@unimi.it (C.M.B.); emma.copelotti@studenti.unimi.it (E.C.); luca.chiesa@unimi.it (L.M.C.); 2 Department of Veterinary Medicine, University of Milan, Via dell’Università 6, 26900 Lodi, Italy; giulio.curone@unimi.it (G.C.); gabriele.brecchia@unimi.it (G.B.); 3 Quality Department, Italian Retail Il Gigante SpA, 20091 Milan, Italy; pessina@ilgigante.net; 4 Department of Food, Environmental and Nutritional Sciences, University of Milan, 20133 Milan, Italy; claudio.arnoldi@unimi.it

**Keywords:** high-pressure treatment, fish quality, fish shelf-life extension, microbiology, sensory traits, metabolomics analysis

## Abstract

High-pressure (HP) treatment could lead to several advantages when applied to fish and seafood since it would affect the extension of the shelf life of this highly perishable food. In this regard, this study aimed to evaluate the effect of high-pressure treatment (500 MPa for 2 min at a temperature of 4 °C) on changes in quality on two different kinds of fresh fish fillets (*Salmo salar* and *Pleuronectes platessa*). Specifically, physico-chemical (VOCs, untargeted metabolomics spectra, pH and color), microbiological (*Enterobacteriaceae*, *Pseudomonas* spp., mesophilic and psychrotrophic bacteria) and sensory traits were evaluated at different days of refrigerated storage. From the results obtained, it is possible to state that the high pressure significantly (*p* ≤ 0.05) reduced microbial growth for each investigated microorganism. Regarding the colorimetric coordinates, no remarkable effects on a* and b* indices were found, while a significant effect (*p* = 0.01) was observed on the colorimetric index L*, making the HP-treated samples lighter than their respective controls. The sensory analysis showed that for the odor attribute, the HP treatment seems to have had a stabilizing action during shelf-life. Moreover, the treated samples obtained a better score than the respective controls (*p* ≤ 0.05). With regards to texture and appearance attributes, the treatment seems to have had a significant (*p* ≤ 0.05) effect, making the treated samples more compact and opaque than controls, therefore resulting in the loss of the characteristics of raw fish for the treated samples. Moreover, from a chemical point of view, HP treatment prevents the development of volatile sulfides and delays the formation of histamine (*p* ≤ 0.05). Very interestingly, the metabolomic approach revealed novel dipeptide markers for the HP procedure.

## 1. Introduction

Fresh fish is a high-quality product with considerable economic importance. It is usually sold vacuum-packed and stored under cold conditions without further treatment [1]. However, seafood is known to be highly perishable with a shelf life of 14 days for a fresh or thawed product. Usually, beyond 7 days of cold storage, the product is considered to be of a lower quality and is frequently sold at reduced cost or discarded [2]. Additionally, the quality of fresh fish is related to the storage conditions and to the different species, as they have different biological and microbiological composition. Therefore, the increasing consumer demand for minimally processed, additive-free and fresh foods with extended shelf-life has triggered the development of non-thermal technologies such as high-pressure processing (HPP) [3].

High-pressure (HP) treatment is a non-thermal and additive-free food preservation technology widely used for highly perishable food products such as meat and fish. The high-pressure technology is able to inactivate pathogen microorganisms, modifying enzymatic activity, reducing desirable compounds losses, thus preserving the food’s freshness and nutritional values [4]. The shelf-life of fresh fish and seafood is short, and processing methods to inhibit spoilage and thereby increase shelf-life would be an advantage [5]. In addition to the microbiological quality parameters, it is also important to document developments of odor, flavor, color, liquid loss, and texture during storage [6]. The HPP food processing system is based on three principles (Le Chatelier’s principle, the principle of microscopic ordering, and the isostatic principle) that lead to minimal physical and chemical changes of treated foods [7]. Low-molecular-weight molecules such as aroma compounds, vitamins, and minerals are rarely affected as such by HP due to the very low compressibility of covalent bonds [8]. On the other hand, macromolecules, such as proteins and starch, can change their native structure during HP in a manner similar to thermal treatments [9,10]. HP treatment is characterized by three processing parameters: temperature (T), pressure (P), and exposure time (t) [9]. In addition, it is known that effects such as discoloration, increased hardness, changes in water-holding capacity, pH variations, lipid oxidation, and protein oxidation, among others, can occur in more intensive treatments [11]. The HP technology working with pressures between 100 and 1000 MPa is able to inactivate different microorganisms [12]. Nevertheless, changes in the microorganism’s morphology caused by HP, might be more or less reversible based on the applied pressure [13]. Then, the efficiency of the HP technology against microorganisms depends on the extent of the pressure, time of pressurization, the temperature of the process, type of microorganism, food type, etc. [14]. Given the different effects that high-pressure treatment can induce in fish fillets, further research is needed to identify the optimal pressure levels concerning different fish [15]. For this reason, this study aimed to evaluate the effect of high-pressure treatment (500 MPa for 2 min at a temperature of 4 °C) on quality changes specifically on physico-chemical, microbiological and sensory traits on two different kinds of fresh fish fillets (*Salmo salar* and *Pleuronectes platessa*). In particular, the effect of HP was evaluated on these two fish species, as the annual Ismea report [16] at the Italian level in 2018 showed an increase in the purchase of fresh or frozen packaged fish, in particular of plaice and cod fillets (+2.6%), while for salmon, EUMOFA reports [17] showed that in 2020 the Italian consumption of salmon grew by 5%, reaching a five-year high in terms of both volume and price (14.92 €/kg, +2% compared to 2018).

## 2. Materials and Methods

### 2.1. Samples and Experimental Design

The salmon (*Salmo salar*) and plaice (*Pleuronectes platessa*) fresh fillets were provided by local fish company and were transported on ice to Food Inspection Laboratory of the Department of Health, Animal Science and Food Safety “Carlo Cantoni”, (University of Milan) within 24 h of deboning. On arrival, the fillets were immediately divided into two groups:

- S_CTRL: salmon fillets untreated in vacuum skin packaging (VSP);
- S_HPP: salmon fillets in VSP and subjected to high-pressure treatment (500 MPa for 2 min at a temperature of 4 °C);
- P_CTRL: plaice fillets untreated in VSP;
- P_HPP: plaice fillets in VSP and subjected to high-pressure treatment (500 MPa for 2 min at a temperature of 4 °C).

Throughout the experiment, the samples were stored at a controlled temperature of 4 °C and analyzed at the following times: on the day of packaging (D0); after three (D3), six (D6), eight (D8), and twelve (D12) days of storage. The entire salmon and plaice fillets weighed approximately 250 g and 100 g, respectively, and at each time point of analysis, three (*n* = 3) samples for each group were randomly removed from storage and subjected to physico-chemical, microbiological, and sensory analyses. VOCs, microbiological, color, pH, and sensory analyses were performed as long as fillets met hygiene parameters (to assess microbiological acceptability, the microbiological results obtained each day of analysis, in terms of Log CFU/g of sample, were evaluated in their entirety); therefore, untreated samples were analyzed up to D8 while treated samples were analyzed up to D12. As for metabolomic analysis, it was carried out only on the day of packaging (D0).

### 2.2. Chemical Analyses

#### 2.2.1. HPLC-Q-Exactive-Orbitrap^®^-MS Metabolomics Analysis

Histamine quantification as an indicator of microbial histamine-decarboxylase activity and the acquisition of untargeted metabolomic spectra was performed using HPLC-Q-Exactive-Orbitrap^®^ high-resolution mass spectrometry followed by Compound Discoverer™ data processing, according to the recently developed strategy for fish muscle we published [18]. All analyses were performed in three biological triplicates. Heatmap (HP) and volcano plot analysis (VP) was performed on the metabolomics data to highlight the differences among fresh and HPP-treated samples.

#### 2.2.2. HS-SPME-GC-MS Analysis for Volatile Substances (VOCs)

The volatiles profile was performed through HS-SPME-GC-MS analysis of volatile organic compounds (VOCs).

All the samples were prepared by weighing exactly 5 g of fish in a 20 mL glass vial along with 100 μL of the internal standard (IS, 4-Methyl-2-pentanone, 2 mg/L in 2-propanol). Five grams were obtained from entire fish fillets previously homogenized in order to select a representative amount of each sample. Each vial was fitted with a cap equipped with a silicon/PTFE septum (Supelco, Bellefonte, PA, USA) and passed in an ultrasonic bath for 10 s at 30 °C., fitted with a cap and equipped with silicon/PTFE septa (Supelco, Bellefonte, PA, USA). At the end of the sample equilibration period (1 h), a StableFlex fiber (Supelco, Bellefonte, PA, USA) conditioned (1.5 h at 280 °C) 50/30 μm Divinylbenzene/Carboxen/polydimethylsiloxane (CAR/PDMS/DVB) was exposed to the sample headspace for extraction (120 min) using an autosampler with a CombiPAL injector system (CTC Analytics, Switzerland). The fiber and extraction time used in this study were selected after the preliminary study. The best adsorption of analyte was obtained using CAR/PDMS/DVB and 120 min as extraction time. The extraction temperature of 25 °C was selected to avoid possible alterations of the matrix (oxidation of some compounds, particularly aldehydes and furans). To keep a constant temperature during analysis, the vials were maintained on a heater plate (CTC Analytics, Zwingen, Switzerland).

GC-MS analysis was performed using a Trace GC Ultra (Thermo-Fisher Scientific, Waltham, MA, USA) gas chromatograph coupled to a Trace DSQII single quadrupole mass spectrometer (MS) (Thermo-Fisher Scientific, Waltham, MA, USA) and equipped with an Rtx-Wax column (30 m; 0.25 mm i.d.; 0.25 μm film thickness, Restek, USA). The oven temperature program was: from 35 °C, hold 8 min, to 60 °C at 4 °C/min, then from 60 °C to 160 °C at 6 °C/min, and finally from 160 °C to 200 °C at 20 °C/min, hold 20 min. Carryover and peaks originating from the fiber were regularly assessed by running blank samples. After each analysis, fibers were immediately thermally desorbed in the GC injector for 5 min at 250 °C to prevent contamination. The injections were performed in splitless mode (8 min). The carrier gas was helium at a constant flow of 1 mL/min. The transfer line to the mass spectrometer was maintained at 230 °C, and the ion source temperature was set at 250 °C. The mass spectra were obtained by using a mass selective detector with the electronic impact at 70 eV, a multiplier voltage of 1456 V, and by collecting the data at rate of 1 scan s^−1^ over the m/z range of 35–350. Compounds were identified by comparing the linear retention indices with the literature data and through the National Institute of Standards and Technology (NIST) MS spectral database, as in previous research [19]. Volatile compounds measurements from each headspace of fish extracts were carried out by peak area normalization (expressed as ppb, internal standard equivalents). All analyses were carried out in triplicate.

### 2.3. Microbiological Anlyses

In order to observe the impact of HP treatment on the microbiological profile, at each analysis time and on three (*n* = 3) samples for each group, the following parameters were evaluated: *Enterobacteriaceae, Pseudomonas* spp., mesophilic and psychrotrophic bacteria. Briefly, the count of *Enterobacteriaceae* was performed using 3M Petrifilm Enterobacteriaceae Count (EB) plates (3M, St. Paul, MN) then incubated at 37 °C for 24 h, while mesophilic and psychrotrophic bacteria were enumerated using 3M Petrifilm Aerobic Count (AC) plates (3M, St. Paul, MN) and the plates were incubated at 30 °C for 48 h and 4 °C for 10 days, respectively. *Pseudomonas* spp. were determined using Cephaloridine Fucidin-Cetrimide selective medium (CFC, OXOID, Basingstoke, Hampshire, UK) and the plates were incubated at 25 °C for 48 h as described by Castrica et al. [20]. The results were reported as Log CFU/g of sample and the analyses were performed in duplicate.

### 2.4. Color and pH Analyses

The evaluation of color, using the CIE L*a*b* color coordinates, was determined by a colorimeter Minolta Croma-Meter CR-400 (Minolta Camera Co., Ltd., Osaka, Japan). The average of 5 measurements of each color parameter was reported. All experiments were performed in duplicate. Moreover, at each time considered, the pH was measured in triplicate using a pH meter equipped with an insertion electrode on 3 fillets per group (Crison pH25, Crison, Barcelona, Spain).

### 2.5. Sensory Analysis

The sensory attributes of salmon and plaice fillets treated and untreated were evaluated by a panel of five experienced judges on each sampling day. The judges were selected from among the laboratory staff and trained in the basic concepts of descriptive analysis and terminology (due to the restrictions caused by the COVID-19 health emergency, it was not possible to recruit people outside the structured staff of the laboratory and in a number greater than five). Both salmon and plaice samples were evaluated on the same day at an interval of one hour. Sensory evaluation of the samples was carried out under controlled environmental conditions (light, temperature, and humidity) in sensory analysis laboratory. Moreover, each judge carried out the evaluation in separate, individual booths. Judges were asked to rate sensory attributes using a nine-point descriptive scale for appearance, odor, and texture, as reported in Table 1 [21]. Single fillets were served on white plastic plates identified by an ID number specific for the control and treated samples.

### 2.6. Statistacal Analysis

VOCs, microbiological, color, pH, and sensory data were tested for normality verification (Shapiro–Wilk). Since the normality assumption was not satisfied, a nonparametric test was applied; specifically, the Mann–Whitney test was used for between-group comparisons and the Wilcoxon signed rank test was used for within-group comparisons during storage days. *p*-values ≤ 0.05 were considered as statistically significant. The data were analyzed with the use of SPSS statistical software, version 26.0 (IBM, Armonk, NY, USA).

## 3. Results and Discussion

### 3.1. VOCs Analysis

**Figure 1:**
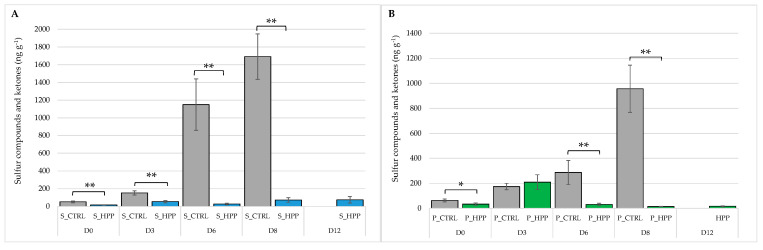
Trend of sulfur compounds and ketones on salmon (S_CTRL and S_HPP; (**A**)) and plaice (P_CTRL and P_HPP; (**B**)) fillets during shelf-life. The bar graphs show mean (*n =* 3) ± standard deviation. Significant effect of groups is highlighted by asterisks: * *p* ≤0.05; ** *p* < 0.01.

Data that have been focused on the examination of the response of global volatilome of fish and seafood to different HP treatments are only occasional in the available literature [22]. The volatile components of salmon and plaice accumulated during the storage practice of fresh fish might be generated/altered by different mechanisms that include lipid oxidation and degradation [23] as well as amino acids/small peptides modifications [24]. For example, a high content of ketones could result from microbially induced lipid oxidation, while sulfur-containing VOCs are the products of methionine and cysteine degradation [25]. Our results indicate that the formation of sulfur-containing compounds (methanthiol, dimethyl sulfate, dimethyl disulfate, dimethyl trisulfate and mercaptoacetone) and ketones (2,3 butanedione and 3-hydroxy-2-butanone) were efficiently prevented by the HP treatment applied (Figure 1A,B).

### 3.2. Untargeted Metabolomics Analysis

**Figure 2:**
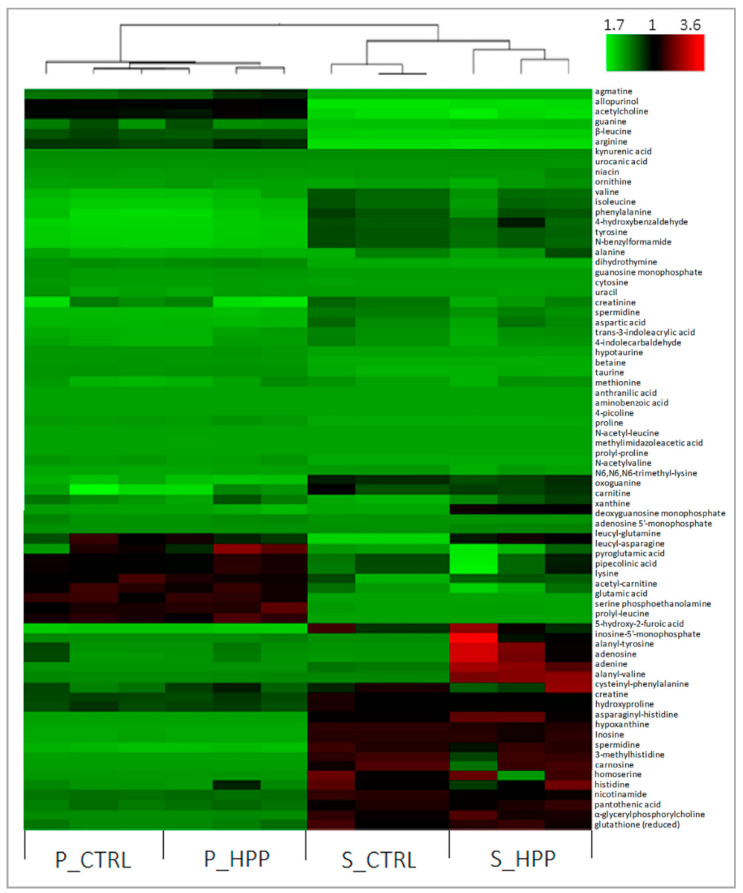
Hierarchical clustering analysis for identification of different metabolites in fish muscle of salmon (S_CTRL and S_HPP) and plaice (P_CTRL and P_HPP) obtained by HPLC-Q-Exactive-Orbitrap^®^-MS untargeted executed in positive mode ionization. Each column in the figure represents a sample (performed in three biological replicate), while each row represents a metabolite, and the color indicates the relative amount of metabolites: red designates that the metabolite is expressed at high levels, and green specifies lower incidence.

**Figure 3:**
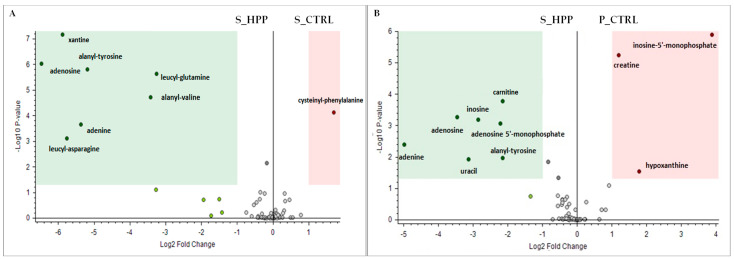
Volcano plot comparison between the relative intensity of chromatographic peak from salmon (S_CTRL and S_HPP; (**A**)) and plaice (P_CTRL and P_HPP; (**B**)) samples. The left region contains up-regulated signal with intensities from HPP-treated samples significantly higher than those from fresh samples and were greater than the upper fold-change (FC) threshold. The right region includes downregulated peaks where the intensities from HP treatment was significantly lower than those from fresh samples and was less than the lower FC threshold; (*p* ≤ 0.05).

The untargeted metabolomic analysis was carried out in order to assess the differences in polar metabolome profile as a function of the HPP treatment compared to the fresh counterparts of the same fish. As shown in Figure 2, 72 metabolites belonging mainly to the pool of free amino acids/small peptides and their metabolites including N-acyl and methyl derivatives, organic acids, nucleotides/nucleosides (adenosine, AMP, IMP, etc.), with the presence of various enzymatic cofactors (e.g., nicotinamide and niacin) were identified. The heat map (Figure 2) clearly demonstrates intra-species differences, regardless of the treatment. The plate samples were reached in lysine and glutamic acid, which was followed by a high concentration of lipid-derived serine-phosphoethanolamine species. The salmon displayed a higher content of histidine, glutathione, and ATP derivates such as inosine and hypoxanthine. Regarding the differences between controls and HP treatment, volcano plot evaluation (Figure 3A,B) confirmed the data published earlier by Huijuan et al. [26] that concerns the modifications of the adenosine-5′-monophosphate (AMP) energetic pathway. A particularly important finding concerns the plate HPP up-regulated AMP level that is followed by simultaneous IMP (inosine-5′-monophosphate) down-regulation. It is known that AMP and IMP are the most prominent flavor-contributing 5′-nucletides that are responsible for the savoriness (umami taste) in some seafood classes [27]. Major routes in IMP production and degradation have been shown to involve two biochemical reactions: (a) deamination of AMP by adenosine monophosphate deaminase (AMPD), situated manly in skeletal muscle; and (b) dephosphorylation of IMP by alkaline (ALP) acid phosphatase (ACP) followed by 5′-nucleotidase activity [26]. These reactions work cooperatively to maintain the balance in IMP content. The fact that HP treatment of plaice decreases the IMP with simultaneous enrichment of degradation products (hypoxanthine and xanthine) points toward the conclusion that high pressure somehow inhibits the AMPD enzymatic activity with no effects on ALP and ACP action. From the VP analysis, it can be noted that there are just a few dipeptides with significant alterations caused by HP processing, while the free amino acids pool basically did not undergo any important variations (Figure 2). This finding is of particular importance as it was published previously that HPP management caused a non-specific shift in the free amino acid profile [28]. Nevertheless, the alanyl-tyrosine was HPP upregulated either in salmon or in plaice, which nominates this dipeptide as a mutual marker for the HPP (500 MPa) processing of salmon and plaice. Alterations in Ala-Tyr concentration are most likely due to denaturation/oxidation events of the protein that would need further clarification. Moreover, the salmon muscle that underwent the HP treatment expressed the downregulation of cysteinyl-phenylalanine with respect to the control samples. Apart from the fact that phenylalanine is one of the most abundant free amino acids in the salmon muscle [18], the mechanisms that decreased the concentration of its dipeptide with cysteine due to HPP manipulation remain to be elucidated.

### 3.3. Microbiological Analysis

**Figure 4:**
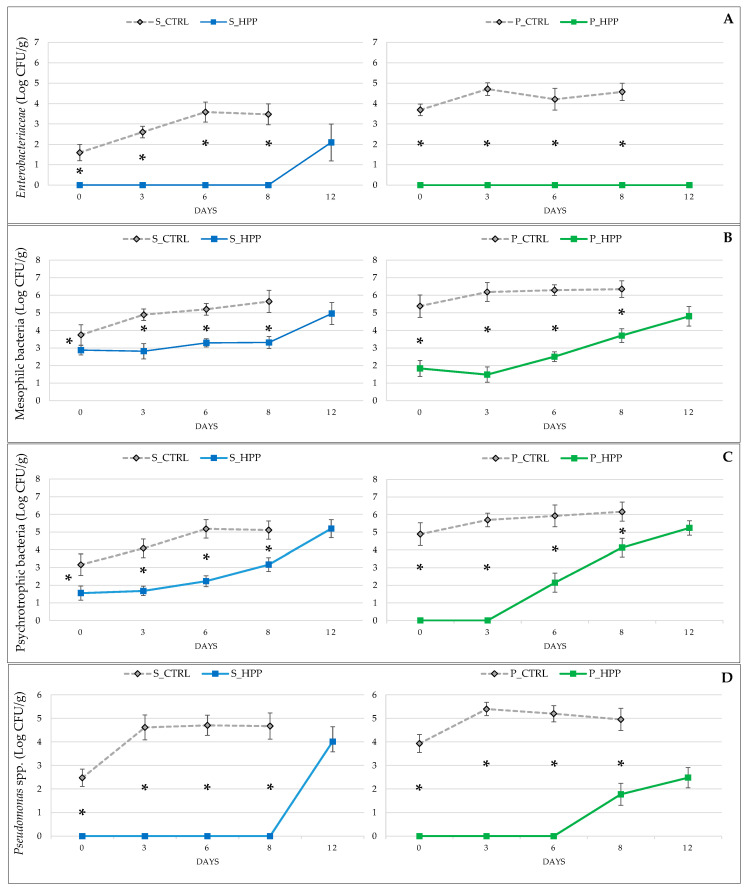
Effects of high-pressure treatment on (**A**) *Enterobacteriaceae*, counts of mesophilic and psychrotrophic bacteria (**B** and **C**, respectively) and *Pseudomonas* spp. (**D**) on salmon (S_CTRL and S_HPP) and plaice (P_CTRL and P_HPP) fillets during shelf-life. The line graphs show mean (*n* = 3) ± standard deviation. Significant effect of groups is highlighted by asterisks: * *p* ≤ 0.05.

**Figure 5:**
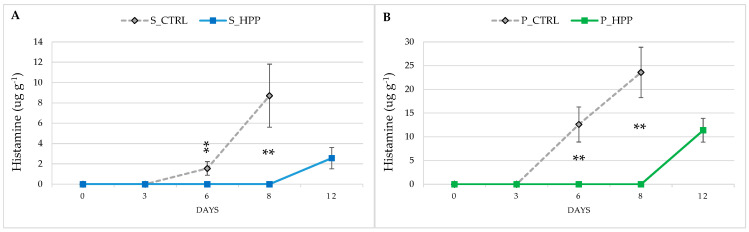
Trend of histamine on salmon (S_CTRL and S_HPP; (**A**)) and plaice (P_CTRL and P_HPP; (**B**)) fillets during shelf-life. The bar graphs show mean (*n* = 3) ± standard deviation. Significant effect of groups is highlighted by asterisks: ** *p <* 0.01.

Microbial growth is the main cause of fish spoilage, which is related to the development of ammoniacal odors typical of spoilage processes. The microbiological results (Figure 4A–D) obtained in this study show that HP treatment has a strong effect on microbial growth in treated salmon and plaice samples. In fact, at all times of analysis and for all parameters investigated in both S_HHP and P_HPP, there was a significant (*p* ≤ 0.05) reduction in microbial growth. The high-pressure treatment was able to maintain the microbiological profile of the HP-treated fish fillets acceptable until the 12th day of storage. Since fish is one the most important single sources of high-quality protein [29], but at the same time has a very short shelf life due to its highly perishable nature, being able to achieve 12 days of commercial storage without obvious microbiological changes represents an opportunity for the industry and fish retail. These microbiological results were supported by histamine evaluation that did not express any increase during the shelf-life of HP-treated fish, except the last point (12th day), where low concentrations were measured, still far below the lower legal limits of 100ug g^−1^ (Regulation EC No. 1441/2007; Figure 5A,B) [30,31]. It is worth noting that the increase in histamine concentration in controls on the sixth and eight day was not followed by any significant alterations of microbiological status. The reason for this might be found in spontaneous proteolysis, which increases as the shelf life progresses. This causes a better availability of histidine and a higher rate of its decarboxylation, causing an increase of histamine concentration. Several authors [32,33,34,35] have highlighted the effect of HP on the inactivation of different types of microorganisms, especially in delaying microbial proliferation, similar to a bacteriostatic power. Yagiz et al. [2] report that pressures of 450 and 600 MPa can reduce the total bacterial count by 4 to 6 Log, while pressure levels of 100 MPa for 30 min appear to induce a significant reduction of the initial level of pseudomonads [1]. Bacteria such as psychrotrophic Gram-negative *Pseudomonas* spp. and H2S-producing bacteria appear to be the most sensitive to HP, and for their inactivation, a treatment at 100 MPa and 5 °C for 30 min is sufficient [1], while *Enterobacteriaceae* and luminescent bacteria are more resistant and a pressure of 300 MPa is necessary to achieve a significant reduction [3].

### 3.4. Color, pH and Sensory Analysis

The results regarding pH were reported in Table 2. The pH, in general, tends to decrease over time and then stabilizes in the final days of storage in all groups until the end of the observation period, except for the S_CTRL group, where it increases significantly until D3 (*p* ≤ 0.05) and then stabilizes. However, no significant effect of HP treatment on pH was evident, in agreement with the findings of Rode and Hovda [15], which show that there are relatively small differences in pH comparing unprocessed and HPP fish. As far as color is concerned, this parameter represents a fundamental aspect for fish, since it is one of the indices for assessing freshness and is often one of the main factors influencing consumers’ purchasing choices [3,36,37]. In our study, in general, over time and for both groups (controls and treated), L* and b* indices tend to increase in the first days of storage and then stabilize at the end. Instead, a* decreases (*p* ≤ 0.05) initially in the initial conservation days in all groups except for the S_CTRL group, where it increases (D0 vs. D3; *p* ≤ 0.05), but again, in all cases, it tends to stabilize at the end of the storage period (Table 2). The discoloration of fish is a frequent phenomenon and is one of the more serious concerns in the seafood industry, often dependent on the activity of micro-organisms in combination with the activities of endogenous enzymes, leading to the development of off-flavor, texture change, discoloration, and other changes typical of fish spoilage [38]. In our study, it is interesting to note that HP treatment has a significant effect on the lightness index; in fact, at each time of analysis, both the S_HPP and P_HPP groups were lighter than their respective controls (*p* = 0.01). Several studies confirm this increase in L* value after high-pressure treatment [21,39,40]. In particular, Matser et al. [39], in agreement with our results, showed that significant increases in the L* index on turbot fillets occurred already with a treatment of 100 MPa for 15 min (L*= 59.1± 0.5 for untreated vs. 64.7± for treated), then increasing pressure and minutes of treatment, the L* index continues to grow until it reaches a maximum value of 82.6 ± 0.6 for samples treated at 200 MPa for 30 min. An increase in the L* index was also shown by Lakshmanan et al. [41] on cold smoked salmon, where minimal color changes occurred with 100 MPa at 30 °C for 10 min and the greatest with a treatment of 300 MPa at 20 °C for 30 min. The intensity with which L* increases seems to be related to the intensity of the treatment (time–pressure–temperature), which could induce the globin and myofibrillar denaturation [1,42,43]. Several authors [1,40,44] have pointed out that an increase in L* induces an increase in the opaqueness in fish muscle. Concerning the a* index, in both treated groups, there is a significant increase (*p* ≤ 0.05) in the first days of storage compared to the controls, but then at D12, these differences disappear. It therefore seems that the high-pressure treatment had an effect that only occurs in the first few days of storage. This result is not in agreement with what is reported in the literature, wherein several authors have reported a decrease in the a* value after high-pressure treatment [11,41]. Finally, with regards to the b* coordinate (index of yellow), no statistically significant differences between the P_CTRL and P_HPP groups were detected at any time of analysis; however, concerning the S_HPP group, there were significant (*p* ≤ 0.05) differences compared to the control group, which disappeared on the eighth day of storage. In this case, the treatment seems to have no influence, a finding which agrees with Oshima et al. [45], who observed no significant changes during storage induced by HPP treatment on the b* index in raw cod and mackerel muscle. In general, however, it can be said that the real effect of HPP on the color change was not fully elucidated, as this depends on many variables (e.g., muscle hydration status, state of myofibrillar and sarcoplasmic proteins: native or denatured) which together have different impacts on color change [46,47]. Indeed, Erkan et al. [21] showed different results from ours and those reported by other authors [1,39], while, regarding the L* index, the cold smoked salmon treated with different combinations of pressure, temperature, and time, specifically: 220, 250 and 330 MPa for 3 °C/5 min, 3 °C/10 min, 7 °C/5 min, 7 °C/10 min, 15 °C/5 min, 15 °C/10 min, 25 °C/5 min and 25 °C/10 min, never showed any significant difference compared to the control, except for treated samples with 220 MPa at 15 °C for 5 min and 15 °C/10 min and 25 °C/5 min, where there is a significant decrease.

In general, with the increase of storage days, there was a trend of decreasing sensory scores in both groups (treated and controls). From the sensory analysis (Table 3), it emerges that between the treated samples and the respective controls at all analysis times there are statistically significant differences for appearance and texture, except for the odor attribute, where the treatment with HP seems to have had a stabilizing action. It is interesting to note that in the P_HPP group, the odor remained stable over time until D3 and then underwent variations that remained within the acceptable range until day D6. Furthermore, the comparison between P_HPP and P_CTRL, always concerning odor, showed significant differences at D3, D6, and D8 (*p* ≤ 0.05), highlighting how the treated sample obtained better sensory scores than the control. The same effect is shown on the S_HPP, where the odor, for the judges, remained stable over time until D6, and again, there are significant differences between S_HPP and S_CTRL at storage days 3, 6, and 8 (*p* ≤ 0.05) with better sensory scores for the samples subjected to high-pressure treatment. It has already been shown that the HP has a strong effect on the elimination of microorganisms, inhibiting the production of biogenic amines, volatile nitrogen, and trimethylamine [32,48]; this could explain the odor stabilization effect and could directly contribute to a better acceptance of the product by the consumer.

Regarding appearance, both salmon and plaice treated with HP were more opaque with a color typical of the cooked product, as also reported by de Oliveira et al. [11], thus losing the shiny color typical of raw fish. This evidence is also consistent with our results obtained by instrumental analysis (L* index). These results are in agreement with the findings of Hurtado et al. [49], who showed that vacuum-packed hake muscle subjected to 400 MPa (three 5-min cycles) at 7 °C had the appearance of cooked muscle, whereas at lower pressures, specifically 200 MPa (three 5-min cycles) at 7 °C, the muscle retained the appearance of raw fish.

It has also emerged that the treatment, in terms of texture, makes the samples more compact and harder compared to the controls, in accordance with what has been shown by different authors [48,50,51]. Yagiz et al. [2] showed an increase in hardness in bluefish muscle pressurized at 100 MPa at room temperature for 30 min, and in Mahi Mahi (*Coryphaena hippurus*) treated at 300 MPa/18.9 °C/15 min. Similarly, Angsupanich et al. [51] noted that the chewiness and hardness of cod, treated at 400 and 600 MPa at room temperature for 20 min increased significantly compared to fresh samples or those treated with lower pressures such as 200 MPa. Lastly, Chéret et al. [52] showed that the hardness of sea bass remained constant during 14 days of storage at 4 degrees after being pressurized at 100 to 300 MPa and 10 °C for 5 min, while changes occurred with pressurization at 400 MPa and 10 °C for 5 min. Most likely, this change in texture is due to the unfolding of actin and sarcoplasmic proteins and the formation of new hydrogen-bonded networks during the treatment [51]. The obtained results lead us to conclude that from a sensory point of view, the fillets pressurized at 500 MPa for 2 min at a temperature of 4 °C lose the appearance of fresh raw fish and take on the typical appearance of cooked fish; this could negatively affect consumer choice. At the same time, however, the treatment allows for an effective shelf-life extension by delaying microbial development and alternative processes; this represents an advantage for the fish industry and retail by contributing to the reduction of surplus and food waste [53], also creating an opportunity for a safe recovery and redistribution to people in food poverty. In particular, we have shown in our results that treatment at 500 MPa certainly has the significant advantage of reducing the microbial count of various microorganisms; on the other hand, it has considerable sensory impacts that could affect the consumer at the time of purchase. For these reasons, testing other combinations of pressure–time–temperature could be useful to extend the use of this technology to the fish sector and for more consumer acceptability. In particular, we have observed from other studies that lower pressures than the one used in our study, e.g., from 100 to 300 MPa, still reduce the initial microbial load significantly with less change in texture and appearance. These different combinations should be studied, investigated, validated, and then proposed to the fish industry for market application. It could also be interesting to propose the application of HP for ready-to-cook fish meals, where the consumer does not demand for the sensory characteristics of fresh fish; on the contrary, a long shelf life of the product is favored.

## 4. Conclusions

HP treatments applied to the fish sector represent a valuable and innovative solution to increase their shelf life and improve their safety. As shown in this study, the treatment has several positive effects, such as: being similar to a bacteriostatic power with regard the bacterial counts and influencing the formation of volatile compounds, thus demonstrating an efficient approach to fresh fish preservation. However, as already pointed out, the effects it has on the texture and appearance could negatively influence the consumer’s choice of purchase since the fish loses the typical characteristics of raw fillet; this could be one of the limitations of use of this technology. For this reason, further studies are needed to correctly modulate the following factors: pressure, time, and temperature in relation to the type of food matrix to be treated, in order to exploit most of the great potential of HP treatment in the fish industry. Moreover, innovative approaches such as metabolomic analysis need to be explored to identify new process markers linked to HP treatment.

## Figures and Tables

**Table 1:** Sensory processing scale.

	9-7 = Good Quality		6.9-4 = Acceptable Quality		3.9-1 = Not Satisfactory Quality	
Attributes	Salmon	Plaice	Salmon	Plaice	Salmon	Plaice
Appearance	Shiny pink with no liquid in the packaging	Shiny white with no liquid in the packaging	Opaque pink with limited amounts of liquid in the packaging	Opaque with limited amounts of liquid in the packaging	Pale pink, opaque; larger presence of liquid in the packaging	White, tending to grey; larger presence of liquid in the packaging
Odour	Typical odour of fresh fish No spoilage		Pungent and metallic odour		Odour of spoilage	
Texture	Soft		Neutral		Hard	

**Table 2:** Effect of high-pressure treatment on pH and color parameters on salmon (S_CTRL and S_HPP) and plaice (P_CTRL and P_HPP) fillets at different storage days (mean ± standard deviation).

Parameters	Groups	Days of Storage				
		Day 0	Day 3	Day 6	Day 8	Day 12
pH	S_CTRL	5.89 ± 0.18 ^Aa^	6.38 ± 0.04 ^Ba^	6.58 ± 0.15 ^Ca^	6.07 ± 0.23 ^ABCa^	-
	S_HPP	6.51 ± 0.20 ^Ab^	6.52 ± 0.20 ^ACa^	6.09 ± 0.18 ^Bb^	6.19 ± 0.14 ^ABCa^	6.44 ± 0.01 ^C^
L*	S_CTRL	44.91 ± 2.57 ^Aa^	58.78 ± 1.96 ^Ba^	54.79 ± 5.18 ^Ba^	48.80 ± 3.02 ^Ca^	-
	S_HPP	70.25 ± 5.06 ^ABb^	71.99 ± 3.38 ^Bb^	73.01 ± 1.63 ^ACb^	73.12 ± 2.78 ^ACb^	73.92 ± 2.10 ^C^
a*	S_CTRL	10.75 ± 0.98 ^Aa^	11.79 ± 1.07 ^Ba^	14.94 ± 0.91 ^ABa^	13.74 ± 3.60 ^Ba^	-
	S_HPP	14.10 ± 3.09 ^Ab^	13.58 ± 1.93 ^BCb^	13.87 ± 1.10 ^ABb^	15.41 ± 2.84 ^Ca^	14.01 ± 1.50 ^D^
b*	S_CTRL	15.62 ± 1.56 ^Aa^	19.97 ± 2.34 ^Ba^	22.54 ± 1.31 ^Ca^	20.47 ± 5.32 ^Ca^	-
	S_HPP	18.56 ± 2.56 ^Ab^	19.66 ± 2.22 ^Ba^	19.05 ± 1.77 ^ACb^	20.86 ± 3.12 ^Ba^	18.71 ± 1.99 ^D^
pH	P_CTRL	6.70 ± 0.05 ^Aa^	6.87 ± 0.22 ^Ba^	6.53 ± 0.02 ^Ca^	6.67 ± 0.02 ^ABCa^	-
	P_HPP	6.62 ± 0.34 ^Aa^	6.79 ± 0.14 ^BCa^	6.83 ± 0.01 ^Bb^	6.48 ± 0.02 ^ABCb^	6.57 ± 0.11 ^C^
L*	P_CTRL	62.14 ± 3.73 ^Aa^	67.25 ± 4.29 ^Ba^	67.15 ± 1.29 ^Ba^	65.34 ± 2.56 ^Ba^	-
	P_HPP	79.50 ± 3.06 ^ABb^	81.08 ± 2.75 ^CDb^	80.91 ± 2.49 ^ACb^	75.12 ± 5.78 ^Eb^	75.31 ± 6.50 ^BDE^
a*	P_CTRL	−1.63 ± 0.50 ^Aa^	−1.43 ± 0.54 ^Ba^	−1.49 ± 1.00 ^ABa^	−1.58 ± 0.47 ^ABa^	-
	P_HPP	−2.77 ± 0.75 ^Ab^	−1.92 ± 0.88 ^BCa^	−2.84 ± 1.12 ^ACb^	−1.46 ± 0.89 ^Ba^	0.11 ± 1.39 ^BC^
b*	P_CTRL	−0.41 ± 1.51 ^Aa^	1.79 ± 2.26 ^Ba^	4.58 ± 1.33 ^Ca^	3.66 ± 1.78 ^Ca^	-
	P_HPP	0.21 ± 1.40 ^Aa^	3.75 ± 3.32 ^Ba^	4.04 ± 0.53 ^Ca^	3.78 ± 3.40B ^Ca^	4.38 ± 2.92 ^C^

Different uppercase letters in the same line indicate significant differences within storage days for each parameter (*p* ≤ 0.05). Different lowercase letters in the same column indicate significant differences between groups for each parameter (*p* ≤ 0.05).

**Table 3:** Sensory changes of salmon (S_CTRL and S_HPP) and plaice (P_CTRL and P_HPP) fillets at different storage days (mean ± standard deviation).

Attributes	Groups	Days of Storage				
		Day 0	Day 3	Day 6	Day 8	Day 12
Appearance	S_CTRL	8.31 ± 0.08 ^Aa^	6.85 ± 0.48 ^Ba^	5.10 ± 0.19 ^Ca^	3.92 ± 0.06 ^Da^	-
	S_HPP	3.18 ± 0.06 ^Ab^	2.16 ± 0.12 ^Bb^	2.07 ± 0.06 ^Bb^	1.93 ± 0.04 ^Cb^	1.55 ± 0.04 ^D^
Odor	S_CTRL	8.87 ± 0.08 ^Aa^	7.15 ± 0.14 ^Ba^	5.64 ± 0.74 ^Ca^	3.81 ± 0.06 ^Da^	-
	S_HPP	8.55 ± 0.31 ^Aa^	8.34 ± 0.07 ^ABb^	8.38 ± 0.06 ^Bb^	4.78 ± 0.07 ^Cb^	4.61 ± 0.04 ^D^
Texture	S_CTRL	8.11 ± 0.11 ^Aa^	7.30 ± 0.16 ^Ba^	6.68 ± 0.50 ^Ca^	3.77 ± 0.06 ^Da^	-
	S_HPP	2.49 ± 0.04 ^Ab^	2.36 ± 0.09 ^Bb^	2.30 ± 0.02 ^Bb^	2.24 ± 0.05 ^Cb^	2.07 ± 0.03 ^D^
Appearance	P_CTRL	5.40 ± 0.25 ^Aa^	5.39 ± 0.55 ^Aa^	5.17 ± 0.16 ^Aa^	2.89 ± 0.12 ^Ba^	-
	P_HPP	2.77 ± 0.14 ^Ab^	1.66 ± 0.45 ^Bb^	1.15 ± 0.03 ^Cb^	1.11 ± 0.12 ^CDb^	1.03 ± 0.04 ^D^
Odor	P_CTRL	8.15 ± 0.36 ^Aa^	7.53 ± 0.34 ^Ba^	3.50 ± 0.30 ^Ca^	1.26 ± 0.15 ^Da^	-
	P_HPP	8.34 ± 0.99 ^Aa^	8.29 ± 0.18 ^Aa^	6.66 ± 0.37 ^Bb^	3.77 ± 0.08 ^Cb^	3.66 ± 0.07 ^D^
Texture	P_CTRL	8.31 ± 0.43 ^Aa^	7.29 ± 0.23 ^Ba^	6.19 ± 0.03 ^Ca^	6.33 ± 0.52 ^Ca^	-
	P_HPP	3.59 ± 0.03 ^Ab^	3.41 ± 0.08 ^Bb^	3.30 ± 013 ^Bb^	1.57 ± 0.08 ^Cb^	1.09 ± 0.005 ^D^

Different uppercase letters in the same line indicate significant differences within storage days for each parameter (*p* ≤ 0.05). Different lowercase letters in the same column indicate significant differences between groups for each parameter (*p* ≤ 0.05).

## Data Availability

Not applicable.
